# The influence of environmental and core temperature on cyclooxygenase and PGE2 in healthy humans

**DOI:** 10.1038/s41598-021-84563-5

**Published:** 2021-03-22

**Authors:** Christopher J. Esh, Bryna C. R. Chrismas, Alexis R. Mauger, Anissa Cherif, John Molphy, Lee Taylor

**Affiliations:** 1 Aspetar–Qatar Orthopaedic and Sports Medicine Hospital, Athlete Health and Performance Research Centre, Aspire Zone, Doha, Qatar; 2 Department of Physical Education, College of Education, Qatar University, Doha, Qatar; 3 Endurance Research Group, School of Sport and Exercise Sciences, University of Kent, Chatham, UK; 4 School of Sport, Exercise and Health Sciences, Loughborough University, Epinal Way, Loughborough, LE11 3TU UK; 5 Sport & Exercise Discipline Group, Faculty of Health, University of Technology Sydney (UTS), Sydney, Australia; 6 Human Performance Research Centre, University of Technology Sydney (UTS), Sydney, Australia

**Keywords:** Enzyme mechanisms, Homeostasis

## Abstract

Whether cyclooxygenase (COX)/prostaglandin E2 (PGE2) thermoregulatory pathways, observed in rodents, present in humans? Participants (n = 9) were exposed to three environments; cold (20 °C), thermoneutral (30 °C) and hot (40 °C) for 120 min. Core (Tc)/skin temperature and thermal perception were recorded every 15 min, with COX/PGE2 concentrations determined at baseline, 60 and 120 min. Linear mixed models identified differences between and within subjects/conditions. Random coefficient models determined relationships between Tc and COX/PGE2. Tc [mean (range)] increased in hot [+ 0.8 (0.4–1.2) °C; p < 0.0001; effect size (ES): 2.9], decreased in cold [− 0.5 (− 0.8 to − 0.2) °C; p < 0.0001; ES 2.6] and was unchanged in thermoneutral [+ 0.1 (− 0.2 to 0.4) °C; p = 0.3502]. A relationship between COX2/PGE2 in cold (p = 0.0012) and cold/thermoneutral [collapsed, condition and time (p = 0.0243)] was seen, with higher PGE2 associated with higher Tc. A within condition relationship between Tc/PGE2 was observed in thermoneutral (p = 0.0202) and cold/thermoneutral [collapsed, condition and time (p = 0.0079)] but not cold (p = 0.0631). The data suggests a thermogenic response of the COX/PGE2 pathway insufficient to defend Tc in cold. Further human in vivo research which manipulates COX/PGE2 bioavailability and participant acclimation/acclimatization are warranted to elucidate the influence of COX/PGE2 on Tc.

## Introduction

Environmental temperature can challenge human homeostasis. Indeed, hot and cold exposures are able to elicit favorable (e.g. hormetic^1,2^) and detrimental^3,4^ acute responses and chronic adaptations. Such exposures (natural or artificial) can be extreme in nature (e.g. sauna, ice water immersion, etc.), yet are used increasingly in clinical^3,5,6^, health^7,8^ and athlete performance (e.g. heat acclimation/acclimatization^1,9^ and rehabilitation^6^ paradigms). Although humans are remarkably tolerant to extremes of temperature through behavioural thermoregulation^10,11^ and acute/chronic physiological^1,2^, biological^12,13^ and psychological^14,15^ responses/adaptations, if the thermoregulatory challenge becomes too great, changes in core temperature (Tc) of ~  ± 2.5 °C can lead to severe and potentially fatal health issues (e.g. heat stroke, cardiac arrhythmias, multi-organ failure, etc.^3,4^) through hypo- and hyper-thermia.

With uncompromised thermoregulation (i.e. without illness or pharmacological compromise) the human body will initiate heat gain/loss mechanisms (including behavioural) in an orderly manner in response to differing environments^16^. Vasoconstriction, extra clothing and shivering in response to cold and vasodilation, seeking shade/a cool environment and sweating in response to heat^10,17,18^. An underlying biochemical mechanism/pathway to Tc regulation has received some attention in recent literature^19–21^ but is yet to be extensively explored or elucidated experimentally (i.e. biochemical analysis) in humans^22,23^. Arachidonic acid oxidation by cyclooxygenase (COX) isoforms (COX1 and COX2) produces prostaglandins, including the pyrogenic mediator prostaglandin E2 (PGE2)^24,25^. In an afebrile state the constitutively expressed COX1 ‘housekeeping’ isoform appears to be the catalyst for PGE2 upregulation^26–28^. In afebrile rodents and other mammals there is evidence of a COX1 splice variant, named COX3, being the catalyst for PGE2 upregulation^20,21,29^. However, in humans the messenger RNA (mRNA) of many COX1 (and COX2) splice variants have been sequenced without determination of any physiological function^30,31^. Indeed, it is estimated ~ 50% of human genes generate multiple mRNA products, often unproductive targets for degradation^32^. Therefore, it would appear that in humans COX1 is the afebrile catalyst for prostaglandin synthesis. In response to pathological states (i.e. febrile) the inducible COX isoform (COX2^24,25^) is stated to be the catalyst for PGE2 upregulation in humans^33,34^ which also results in thermogenesis and a Tc increase (i.e. fever). There is currently no human data to support an afebrile COX1/PGE2 thermogenic pathway (i.e. Tc maintenance/increase^35^) however, a reduction in PGE2 in rodents is synonymous with Tc reduction^20,21,36^. Following acetaminophen [paracetamol (ACT)] administration, a potent in vivo COX/PGE2 inhibitor in humans^37^ and rodents^20,21,36^, a reduction in Tc (up to 3.9 °C) was simultaneous with up to 96% reductions in PGE2 in afebrile rodents housed below their thermoneutral zone (TNZ^20,21,36^); “*the TNZ is a range of ambient temperature at which temperature regulation is achieved only by control of sensible heat loss, i.e., without regulatory changes in metabolic heat production or evaporative heat loss.”*^38^. Although similar ACT mediated (dose: 20 mg kg^−1^ lean body mass^−1^) Tc responses have been observed in humans (reduced Tc by up to 0.57 °C; a response absent without ACT ingestion) when exposed to conditions below their TNZ [10 and 20 °C 40% relative humidity (RH)^22,23^], they were not supplemented by biochemical analysis of COX and PGE2, thus this pathways implication remains unclear in humans^31^.

The evidence (rodents^20,21,36^) and provisional data/discussion (humans^19,22,23^) provides a plausible hypothesis that the COX/PGE2 pathway may influence Tc regulation in humans. Thus, this study will explore the COX/PGE2 pathway (COX1, COX2 and PGE2 concentrations) in response to acute exposure to different environmental conditions (cold, hot and thermoneutral) that challenge or maintain Tc. Indeed, deductive research designs have been called for^35^ and are thus required to elucidate the influence of COX/PGE2 on human Tc regulation. It is hypothesized that rectal temperature (Trec) will: (i) significantly increase in a hot environment; (ii) remain stable in a cold (as seen elsewhere^22,23^) and (iii) thermoneutral environment. Further, concentrations of COX and PGE2 will be implicated in Tc changes, specifically: (iv) COX1 concentrations will decrease (i.e. catalyzed) in the cold environment (where Trec will be challenged yet defended and remain stable) and remain stable in the thermoneutral (where Trec will remain stable) and hot (where Trec will increase); (v) COX2 will remain stable across all conditions in the absence of a febrile stimulus; (vi) PGE2 concentration will increase in the cold but remain stable in the thermoneutral and hot environment.

## Methods

All study procedures were approved by the Anti-Doping Lab Qatar (ADLQ) Institutional Review Board (F2017000234), in the spirit of the Helsinki Declaration and executed within an Orthopaedic and Sports Medicine hospital in accordance with ADLQ guidelines and regulations. Nine healthy males (30 ± 3.0 y, 1.80 ± 0.06 m, 83.0 ± 6.25 kg, body fat % 19.1 ± 4.56) were recruited and voluntarily signed an informed consent form prior to taking part.

A Tc null zone where there is no sweating or shivering has been observed^39^ indicating that a ± 0.3 °C change in Tc is physiologically meaningful (i.e. the induction of significant heat gain/loss mechanisms). A ≥ 0.3 °C change in Tc determined if the environmental conditions significantly disrupted homeostasis. Based on this data a power calculation (G*Power Software Version 3.0.10, Henrich University, Dusseldorf, Germany) was performed for changes in Tc; a sample size of six was required to achieve a statistical power of 0.90. Nine participants were recruited to ensure six full datasets. Normative values or experimental data (including environmental challenges) of COX1, COX2 and PGE2 concentrations, from robust experimental designs, are not available in vivo in humans. Power calculations were therefore not feasible for bio-chemical outcome measures.

This study employed a randomized repeated measures experimental design with three experimental conditions [Cold (COLD), Thermoneutral (TN) and hot (HOT)] and laboratory visits. The COLD condition was determined as ‘cold’, from a thermoregulatory perspective because 20 °C is below the human TNZ therefore, heat retention/generation is required to maintain a homeostatic Tc^40^. Each trial was separated by at least 48 h.

### Experimental controls

Participants did not ingest (confirmed verbally on arrival to each laboratory visit) any over-the-counter or prescription drugs in the 48 h prior to testing, thus specifically controlling for the vast array of drug formulations [e.g. ACT and non-steroidal anti-inflammatory drugs (NSAID)] that target COX isoforms and inhibit the production of prostaglandins in vivo (e.g. PGE2^37,41^). Additionally, they refrained from caffeine intake and exercise in the 24 h prior to testing. Participants arrived at the research laboratory fasted overnight between 08:00 and 10:00 a.m.; timing was kept constant following the first visit to avoid any circadian influence on Tc between trials^42^. Testing only commenced if participants presented with a Tc ≤ 37.5 °C (no participants were excluded for a high baseline Tc).

### Procedures

**Figure 1:**
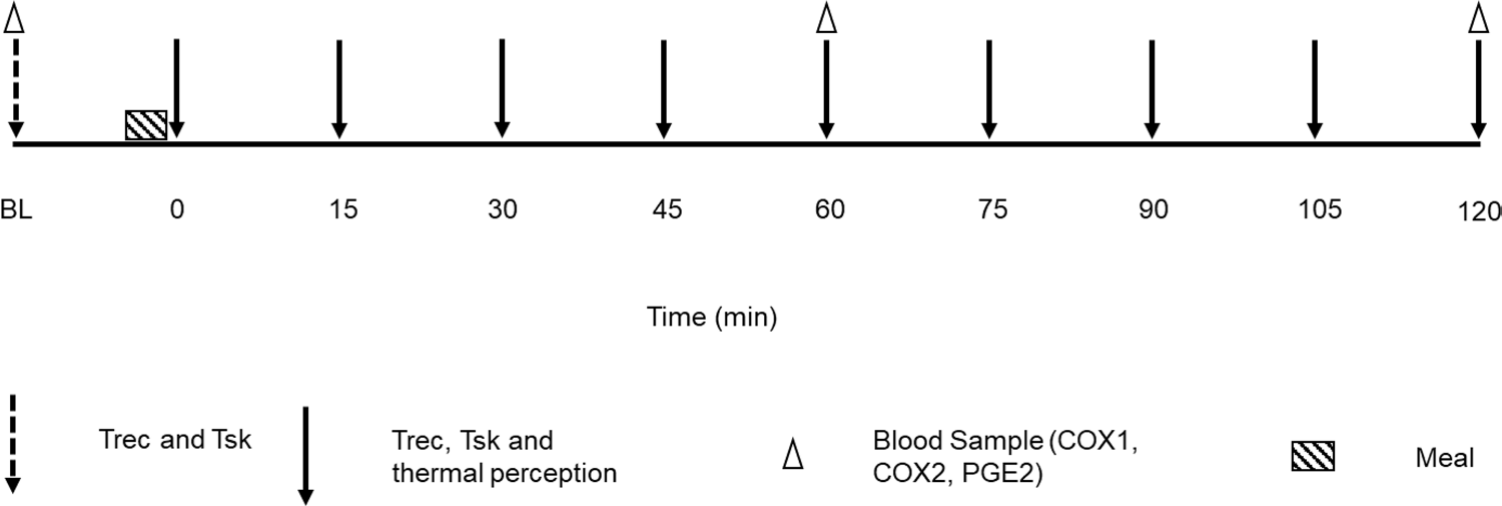
Schematic of experimental procedures. Abbreviations: core temperature (Trec); skin temperature (Tsk); cyclooxygenase (COX); Prostaglandin E2 (PGE2).

Prior to experimental visits body fat % was assessed via dual energy X-ray absorptiometry [DEXA (Lunar iDXA, GE Healthcare, Chicago, USA)] conducted and analyzed by the Aspetar radiology department. Upon arrival at the research laboratory participants had their height and weight measured (first visit only; SECA column scales with telescopic height, Hamburg, Germany) and were instrumented with rectal and skin thermistors. Participants were asked to sit for 10 min in the clothes they arrived in to obtain a baseline Tc. They entered the environmental chamber (Guangdong Sanwood Technology Corporation, Guangdong, China) where a cannula (Vasofix Safety, B Braun, Sheffield, UK) was then inserted and a blood sample taken (for blood sample timings see Fig. 1). A standardized meal was then provided [50 g cornflakes (Kellogg’s, Michigan, USA; 42 g carbohydrates, 0.5 g fat, 3.5 g protein), 250 mL full fat milk (Baladna, Al Khor, Qatar; 11.3 g carbohydrates, 7.5 g fat, 7.5 g protein)]. Once the meal was consumed the 120 min experimental period began.

### Environmental conditions

Participants were exposed to three randomized environmental conditions on separate occasions for 120 min (COLD: 20 °C 40% RH; TN: 30 °C 40% RH and HOT: 40 °C 40% RH). Environmental temperature was statistically significantly different between conditions (p < 0.001), whilst humidity was not (p = 0.438) and within condition environmental conditions presented non-significant minimal variation (mean ± standard deviation: COLD: 20.07 ± 0.11 °C, 40.20 ± 0.53% RH; TN: 29.82 ± 0.19 °C, 40.53 ± 0.80% RH; HOT: 39.94 ± 0.11 °C, 40.21 ± 0.28% RH). In COLD participants were fully clothed (t-shirt, trousers, jacket and socks) for the first hour before they removed their jacket and trousers and remained for the final hour in shorts and socks only. In TN and HOT only shorts and socks were worn for the duration.

### Rectal and skin temperature

Tc data (due to equipment malfunction Tc data is only available for n = 8) was obtained via a rectal thermistor (MRB Rectal Probe, Ellab, Hillerød, Denmark) connected to a medical precision thermometer (DM852 Thermometer, Ellab, Hillerød, Denmark). Participants self-inserted the rectal thermistor 15 cm beyond the anal sphincter upon arrival. To accurately reflect the deep body temperature measurement site used in this study Trec will be used when referring to Tc data from this study^43^. Skin temperature (Tsk) was measured via skin thermistors (iButton Hygrochron Temperature/Humidity Logger, Maxim Integrated, San Jose, USA) that were placed upon four sites (calf, thigh, chest and triceps^44^). Each thermistor recorded separately (n = 7 in the COLD due to iButton thermistors malfunctioning, i.e. not recording data). Upon cessation of each testing day, the data from each thermistor was downloaded to the ibutton data logger (iButton Hygrochron Temperature/Humidity Logger, Maxim Integrated, San Jose, USA). The Ramanathan^44^ formula was used to calculate Tsk (for Trec and Tsk data recording timings see Fig. 1).

### Thermal perception

Thermal perceptions were recorded (for timings see Fig. 1) via visual analogue scales (VAS). Thermal sensation (TS) a scale from 1 to 7 (cold–hot), thermal comfort (TC) on a scale from 1 to 7 (too cool–much too warm) and shivering (SHV) on a scale from 1 to 4 (not at all shivering–vigorously shivering).

### Blood sampling

Blood samples (for timings see Fig. 1) were obtained via cannulation of the antecubital vein. Vacutainer tubes (SST II Advance, BD Vacutainer, Plymouth, UK) were inverted as per manufacturer guidance and left to rest upright for 30 min before samples were spun at 1500×*g* for 10 min in a centrifuge (Hermle Labortechnik GmbH, Wehingen, Germany). Serum supernatant was then isolated, aliquoted and stored at − 80 °C until required for analysis.

### Biochemical analysis

All biochemical markers (COX1, COX2, PGE2) were analyzed by the lead investigator via commercially available enzyme linked immunosorbent assays [ELISA (Cloud-Clone, Texas, USA)] and measured using a Tecan Sunrise Plate Reader (Tecan Group Ltd, Mannedorf, Switzerland). To minimize inter-assay variation, samples from each participant were analyzed on the same ELISA plate. Inter-assay coefficient of variation (CV) for each marker (COX2: 3.78%, PGE2: 6.21%) was less than those stated by the manufacturer [manufacturer stated CV < 10% (intra-assay) and CV < 12% (inter-assay)]. All ELISA analysis was completed as per manufacturer instruction and values adjusted for any observed plasma volume changes across time^45^. Haematocrit concentrations were measured to determine plasma volume^45,46^, the percentage change in plasma volume was added to or subtracted from the absolute concentration of the biochemical marker.

### Statistical analysis

Linear mixed models (IBM-SPSS Statistics for Windows, Version 21, Armonk, NY) were used to determine any differences (fixed effects model) and relationships (random coefficient model) between and within conditions across time. This type of analysis was preferred as it allows for missing data, and can model between-subject variability^47^, reveal the existence and size of causal effect heterogeneity^48^ and determine differences between/within groups^49^. The most appropriate model was chosen using the smallest Hurvich and Tsai’s criterion (AICC) in accordance with the principal of parsimony. Prior to any inferential statistics, descriptive statistics were checked for normality using quantile–quantile plots, and deemed acceptable. Normality and homogeneity of variance of the predicted values and residuals for all variables were checked using scatter plots. Relationships between (i) Trec and COX2, (ii) Trec and PGE2 and (iii) COX2 and PGE2 were assessed; with (i) all data collapsed (condition and time), (ii) collapsed by condition, (iii) collapsed by time and (iv) within condition at each time point. Trec data is only available for 8 participants. Relationships between Trec and COX/PGE2 were therefore determined within 8 participants. COX/PGE2 relationships were determined using the full available dataset (n = 9). Hedges’ *g* effect size (ES) was calculated and interpreted for differences as ‘trivial’ (< 0.20), ‘small’ (0.20–0.59), ‘moderate’ (0.6–1.19), ‘large’ (1.20–1.99) or ‘very large’ (> 2.0). Step-up Hommel^50^ adjusted *post-hoc* pairwise comparison was calculated for each measure if a significant main effect and/or interaction effect was present. Two-tailed statistical significance was accepted at p ≤ 0.05.

## Results

All comparisons are to baseline and expressed as delta (Δ) changes. The presence and size of differences and relationships from the model were not changed whether the raw or Δ data were used. Some raw data will be presented for descriptive purposes. Data will be presented as mean (range) unless stated otherwise.

### Core temperature, COX and PGE2 response to environmental temperature

**Figure 2:**
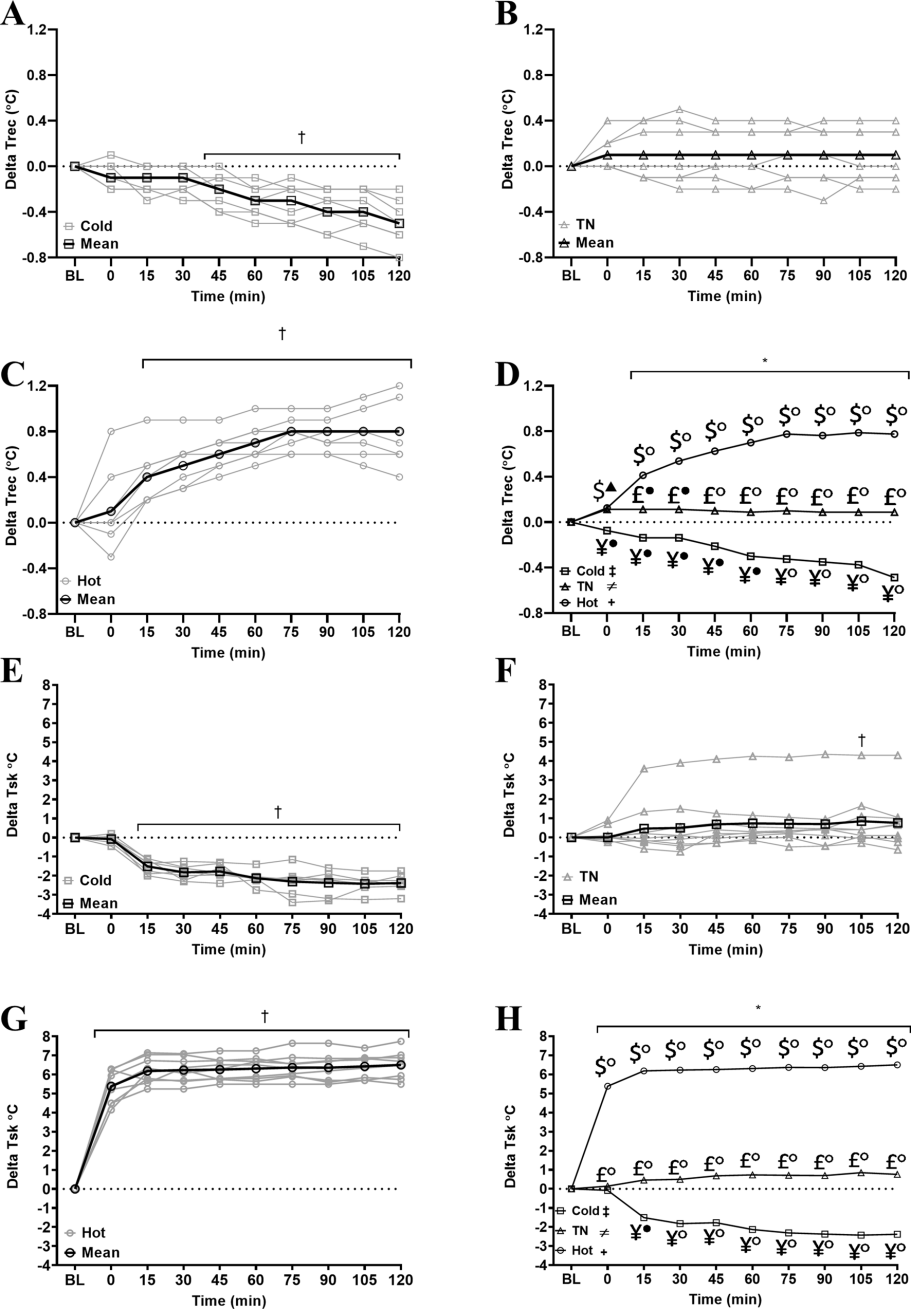
(**A**–**D**) Delta Trec response to environment (n = 8). (**A**) Individual Trec response to a cold environment (20 °C 40% RH). (**B**) Individual Trec response to a temperate environment (30 °C 40% RH). (**C**): Individual Trec response to a hot environment (40 °C 40% RH). (**D**) Mean (no error bars shown for the purposes of clarity) Trec response to all environments. Significant main effect of condition (p < 0.001): ^+^Hot vs TN (p < 0.001); ^‡^Hot vs Cold (p < 0.001); ^≠^TN vs Cold (p < 0.001). ^*^Significant main effect of time (p = 0.01). ^†^Significant condition * time interaction (p < 0.001). Significant interaction between conditions: ^£^Hot vs TN; ^$^Hot vs Cold; ^¥^TN vs Cold. (**E**–**H**) Delta Tsk response to Environment. (**E**) Individual (n = 9) Tsk response to a cold environment (20 °C 40% RH). (**F**) Individual (n = 9) Tsk response to a temperate environment (30 °C 40% RH). (**G**) Individual (n = 7) Tsk response to a hot environment (40 °C 40% RH). NB: statistical model was run with and without the visual outlier in the TN condition and there was no change in statistical analysis outcomes. (**H**) Mean (no error bars shown for the purposes of clarity) Tsk response to all environments. Significant main effect of condition (p < 0.001): ^+^Hot vs TN (p < 0.001); ^‡^Hot vs Cold (p < 0.001); ^≠^TN vs Cold (p < 0.001). ^*^Significant main effect of time (p < 0.001). ^†^Significant condition * time interaction (p < 0.001). Significant interaction between conditions: ^$^Hot vs Cold; ^¥^TNZ vs Cold. Symbols are used to denote the ES (e.g. (filled square) ‘trivial’, (open square) ‘small’, (filled triangle) ‘moderate’, (filled circle) ‘large’, (open circle) ‘very large’).

Trec (n = 8; all conditions) increased in HOT [+ 0.8 (0.4–1.2) °C; p < 0.0001; ES: 2.9, ‘very large’] and decreased in COLD [− 0.5 (− 0.8 to − 0.2) °C; p < 0.0001; ES: 2.6, ‘very large’] and was unchanged in TN [+ 0.1 (− 0.2 to 0.4) °C; p = 0.3502]. Figure 2 and Table 1 display all main effects, interactions and ES.

**Table 1:** Mean differences in Trec (°C) compared to baseline and between condition interactions.

n = 8	Hot	TN	Cold		Hot vs TN	Hot vs cold	TN vs cold
BL	36.9 (36.5–37)	36.8 (36.4 to 37.2)	36.9 (36.6 to 37.3)	P value	0.4519	1.0000	0.4519
				ES	0.36^□^	0.0^■^	0.29^□^
				CI (95%)	− 0.141 to 0.316	− 0.229 to 0.229	− 0.141 to 0.316
0	0.1 (− 0.1 to 0.8)	0.1 (0.0 to 0.4)	− 0.1 (− 0.2 to 0.0)	P value	0.8937	***0.0335***	***0.0461***
				ES	0.05^■^	***0.76***^▲^	***1.41***^●^
				CI (95%)	− 0.17 to 0.2	***− 0.38 to − 0.02***	***− 0.37 to 0.0***
15	0.4 (0.2–0.9)	0.1 (− 0.1 to 0.4)	− 0.1 (− 0.3 to 0.0)	P value	***0.0015***	** < 0.0001**	***0.0081***
				ES	***1.24***^●^	***2.87***^○^	***1.36***^●^
				CI (95%)	***0.116–0.484***	***− 0.73 to − 0.37***	***− 0.43 to − 0.07***
30	0.5 (0.3–0.9)	0.1 (− 0.2 to 0.5)	− 0.1 (− 0.3 to 0.0)	P value	** < 0.0001**	** < 0.0001**	***0.0081***
				ES	***1.75***^●^	***4.02***^○^	***1.20***^●^
				CI (95%)	***0.12–0.48***	***− 0.86 to − 0.49***	***− 0.43 to − 0.07***
45	0.6 (0.4–0.9)	0.1 (− 0.2 to 0.4)	− 0.2 (− 0.4 to 0.0)	P value	** < 0.0001**	** < 0.0001**	***0.0001***
				ES	***2.66***^○^	***5.25***^○^	***1.63***^●^
				CI (95%)	***0.34–0.71***	***− 1.02 to − 0.65***	***− 0.5 to− 0.13***
60	0.7 (0.5–1.0)	0.1 (− 0.2 to 0.4)	− 0.3 (− 0.5 to − 0.1)	P value	** < 0.0001**	** < 0.0001**	** < 0.0001**
				ES	***2.95***^○^	***6.51***^○^	***1.98***^●^
				CI (95%)	***0.43–0.8***	***− 1.18 to − 0.82***	***− 0.57 to − 0.2***
75	0.8 (0.6–1.0)	0.1 (− 0.2 to 0.4)	− 0.3 (− 0.5 to − 0.1)	P value	** < 0.0001**	** < 0.0001**	** < 0.0001**
				ES	***3.49***^○^	***6.82***^○^	***2.07***^○^
				CI (95%)	***0.49–0.86***	***− 1.28 to − 0.92***	***− 0.61 to − 0.24***
90	0.8 (0.6–1.0)	0.1 (− 0.3 to 0.4)	− 0.4 (− 0.6 to − 0.2)	P value	** < 0.0001**	** < 0.0001**	** < 0.0001**
				ES	***3.25***^○^	***6.81***^○^	***2.00***^○^
				CI (95%)	***0.49–0.86***	***− 1.3 to − 0.93***	***− 0.62 to − 0.25***
105	0.8 (0.5–1.1)	0.1 (− 0.2 to 0.4)	− 0.4 (− 0.7 to − 0.2)	P value	** < 0.0001**	** < 0.0001**	** < 0.0001**
				ES	***3.17***^○^	***5.84***^○^	***2.16***^○^
				CI (95%)	***0.52–0.88***	***− 1.35 to − 0.98***	***− 0.65 to − 0.28***
120	0.8 (0.4–1.2)	0.1 (− 0.2 to 0.4)	− 0.5 (− 0.8 to − 0.2)	P value	** < 0.0001**	** < 0.0001**	** < 0.0001**
				ES	***2.67***^○^	***5.22***^○^	***2.65***^○^
				CI (95%)	***0.5–0.87***	***− 1.45 to − 1.08***	***− 0.76 to − 0.39***

Values expressed as mean (range). Raw BL values presented for context. Bold and italic text denotes a significant finding. Symbols are used to denote the ES (e.g. ^■^‘trivial’, ^□^‘small’, ^▲^‘moderate’, ^●^‘large’, ^○^‘very large’).

**Figure 3:**
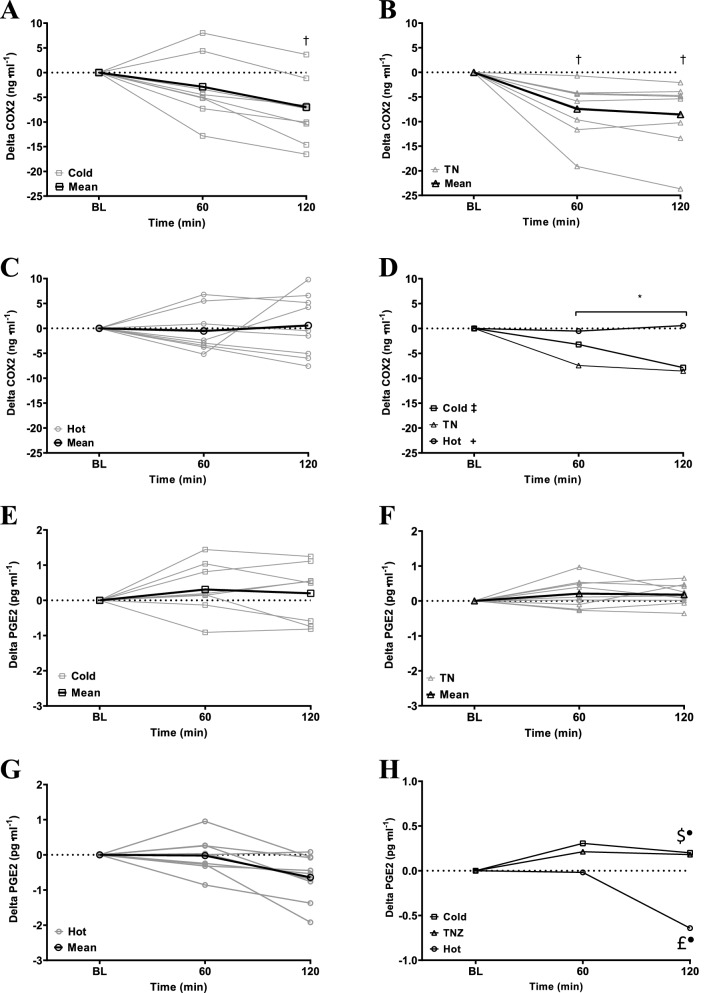
(**A**–**D**) Delta COX2 response to environment (n = 9). (**A**) Individual COX2 response to a cold environment (20 °C 40% RH). (**B**) Individual COX2 response to a temperate environment (30 °C 40% RH). (**C**) Individual COX2 response to a hot environment (40 °C 40% RH). (**D**) Mean (no error bars shown for the purposes of clarity) COX2 response to all environments. Significant main effect of condition (p = 0.001): ^+^Hot vs TN (p < 0.001); ^‡^Hot vs Cold (p = 0.015). ^*^Significant main effect of time (p < 0.01). ^†^Significant condition * time interaction (p = 0.04). ^+^Hot vs TN; ^‡^Hot vs Cold; ^≠^TN vs Cold. *Significant main effect of time (p < 0.001). ^†^Significant condition * time interaction (p < 0.001). Significant interaction between conditions: ^£^Hot vs TN; ^$^Hot vs Cold; ^¥^TN vs Cold. (**E**–**H**) Delta PGE2 response to Environment (n = 9). (**E**) Individual PGE2 response to a cold environment (20 °C 40% RH). (**F**) Individual PGE2 response to a temperate environment (30 °C 40% RH). (**G**) Individual PGE2 response to a hot environment (40 °C 40% RH). (**H**) Mean (no error bars shown for the purposes of clarity) PGE2 response to all environments. Significant main effect of condition (p = 0.006): ^+^Hot vs TN (p = 0.008); ^‡^Hot vs Cold (p = 0.004). Significant interaction between conditions: ^£^Hot vs TN; ^$^Hot vs Cold. Symbols are used to denote the ES (e.g. (filled square) ‘trivial’, (open square) ‘small’, (filled triangle) ‘moderate’, (filled circle) ‘large’, (open circle) ‘very large’).

**Figure 4:**
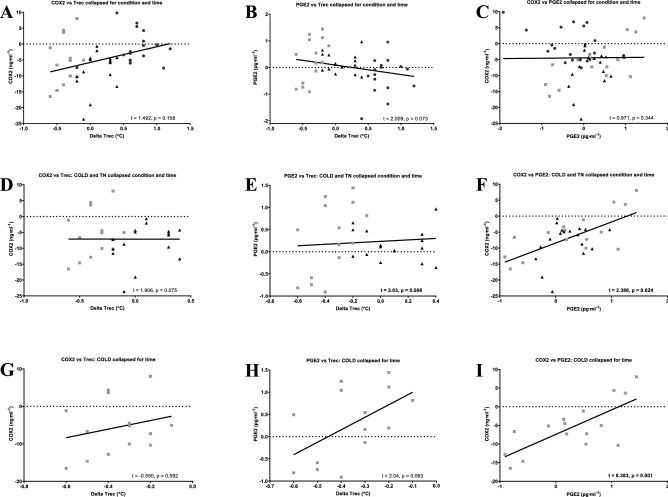
Random coefficient models: the relationships across all conditions and the analysis including the COLD condition are presented (n = 8). Significance of relationship is detailed in each panel; significant relationships are highlighted in bold and italicized. (**A**) COX2 vs Trec collapsed by condition and time. (**B**) PGE2 vs Trec collapsed by condition and time. (**C**) COX2 vs PGE2 collapsed by condition and time. (**D**) COX2 vs Trec: COLD and TN collapsed by condition and time. (**E**) PGE2 vs Trec: COLD and TN collapsed by condition and time. (**F**) COX2 vs PGE2: COLD and TN collapsed by condition and time. (**G**) COX2 vs Trec: COLD collapsed by time. (**H**) PGE2 vs Trec: COLD collapsed by time. (**I**) COX2 vs PGE2: COLD collapsed by time.

COX1 (n = 9) was unable to be detected in the serum with all values below the minimum sensitivity of the ELISA kit (0.225 ng ml^−1^). Normative values for serum COX1 in humans are not available in the literature to the authors knowledge. COX2 (n = 9, see Fig. 3 and Table 2) decreased in the COLD [− 7.0 (− 16.52 to 3.65) ng ml^−1^; p = 0.0033; ES: 1.0, ‘moderate’] and TN [− 8.55 (23.65 to − 2.08) ng ml^−1^; p = 0.0004; ES: 1.3, ‘large’] but was unchanged in HOT (+ 0.58 (− 7.59 to 9.80) ng ml^−1^; p = 0.8032). PGE2 (n = 9, see Fig. 3 and Table 2) did not change across time in any condition (p = 0.1452). Random coefficient models (see Table 3) identified relationships between COX2 and PGE2 (n = 9) in COLD (p = 0.0012) and COLD/TN [collapsed by condition and time (p = 0.0243)]. A within condition relationship between Tc and PGE2 (n = 8) was evident in COLD/TN [collapsed by condition and time (p = 0.0079)] but not COLD (p = 0.0631). Higher PGE2 concentrations were associated with higher Tc (see Fig. 4, panel H). There was no relationship between COX2 and Tc (n = 8) across all models [HOT (p = 0.0986); TNZ (p = 0.5935); COLD (p = 0.5917); COLD/TN collapsed for condition and time (p = 0.0749)].

**Table 2:** Differences in COX2 and PGE2 concentration compared to baseline and between condition interactions.

n = 9	Hot	TN	Cold		Hot vs TN	Hot vs cold	TN vs cold
**COX2 (ng/ml)**							
BL	31.07 (20.44 to 45.66)	38.27 (27.99 to 68.66)	37.22 (28.15 to 49.27)	P value	0.2573	0.0571	0.4308
				ES	0.55^□^	0.78^▲^	0.08^■^
				CI (95%)	− 14.86 to 0.45	− 14.04 to 1.74	− 6.84 to 8.94
60	− 0.53 (− 51.8 to 6.80)	− 7.45 (− 19.11 to − 0.71)	− 2.87 (− 12.82 to 8.02)	P value	***0.0036***	0.312	0.0502
				ES	***1.35***^●^	0.42^□^	0.38^□^
				CI (95%)	***2.34 to 11.50***	− 2.24 to 6.92	− 9.16 to 0.0
120	0.58 (− 7.59 to 9.80)	− 8.55 (− 23.65 to − 2.08)	− 7.00 (− 16.52 to 3.65)	P value	***0.0002***	***0.0015***	0.5001
				ES	***1.36***^●^	***1.12***^●^	0.22^□^
				CI (95%)	***4.54 to 13.71***	***2.99–12.16***	− 6.14 to 3.03
**PGE2 (pg/ml)**							
BL	9.63 (8.44 to 10.46)	9.00 (8.02 to 9.82)	8.54 (7.29 to 10.02)	P value	0.0736	***0.0031***	0.1962
				ES	0.86^▲^	***1.15***^▲^	0.48^□^
				CI (95%)	− 0.62 to 1.32	***0.383–1.81***	− 0.25 to 1.18
60	− 0.02 (− 0.86 to 0.95)	0.21 (− 0.27 to 0.97)	0.31 (− 0.94 to 1.44)	P value	0.3075	0.1528	0.6777
				ES	0.48^□^	0.51^□^	0.16^■^
				CI (95%)	− 0.68 to 0.22	− 0.77 to 0.12	− 0.54 to 0.35
120	− 0.6 (− 1.92 to 0.08)	0.18 (− 0.36 to 0.65)	0.20 (− 0.82 to 1.25)	P value	***0.0005***	***0.0003***	0.9307
				ES	***1.54***^●^	***1.12***^●^	0.03^■^
				CI (95%)	***− 1.27 to − 0.38***	***− 1.29 to − 0.4***	− 0.47 to 0.43

Values expressed as mean (range). Raw BL values presented for context. Bold and italic text denotes a significant finding. Symbols are used to denote the ES (e.g. ^■^‘trivial’, ^□^‘small’, ^▲^‘moderate’, ^●^‘large’, ^○^‘very large’).

**Table 3:** Results from random coefficient model analysis.

	COX2/Tc (n = 8)		PGE2/Tc (n = 8)		COX2/PGE2 (n = 9)	
	Random coefficient	Time	Random coefficient	Time	Random coefficient	Time
**All conditions**						
P value	0.1585	** < 0.0001**	0.0730	** < 0.0001**	0.3437	0.2838
T value	1.492	***6.807***	2.009	***9.479***	0.971	1.10
Estimate	0.003	***0.12***	0.38	***0.14***	0.01	0.096
CI (95%)	− 0.002 to 0.009	***0.08 to 0.15***	− 0.004 to 0.08	***− 0.11 to 0.17***	− 0.01 to 0.03	− 0.09 to 0.28
**COLD vs. TN**						
P value	0.0749	0.9985	***0.0079***	***0.0300***	***0.0243***	0.9468
T value	1.906	0.002	***3.03***	***2.331***	***2.388***	− 0.067
Estimate	0.008	0.00004	***0.122***	***0.09***	***0.03***	− 0.007
CI (95%)	− 0.0009 to 0.17	− 0.04 to 0.04	***0.37 to 0.21***	***0.009 to 0.17***	***0.003 to 0.05***	− 0.23 to 0.21
**COLD**						
P value	0.5917	***0.0378***	0.0631	***0.0372***	***0.0012***	0.1359
T value	− 0.550	***− 2.313***	2.04	***− 2.338***	***8.383***	1.606
Estimate	− 0.004	***− 0.20***	0.098	***− 0.17***	***0.10***	0.29
CI (95%)	− 0.017 to 0.01	***− 0.39 to − 0.01***	− 0.006 to 0.20	***− 0.33 to − 0.01***	***0.05 to 0.14***	− 0.11 to 0.68
**TN**						
P value	0.5935	0.8256	***0.0202***	0.8901	0.1625	0.9620
T value	0.547	0.228	***3.111***	0.144	1.469	− 0.048
Estimate	0.005	0.007	***0.17***	0.004	0.21	− 0.008
CI (95%)	− 0.16 to 0.03	− 0.06 to 0.08	***0.04 to 0.30***	− 0.06 to 0.07	− 0.01 to 0.05	− 0.36 to 0.35
**HOT**						
P value	0.0986	0.0985	0.1506	0.0709	0.0619	***0.0077***
T value	− 1.861	1.936	1.560	2.021	− 2.039	***− 3.580***
Estimate	− 0.01	0.095	0.10	0.14	− 0.047	***− 0.57***
CI (95%)	− 0.31 to 0.003	− 0.02 to 0.21	− 0.44 to 0.25	− 0.01 to 0.30	− 0.09 to 0.003	***− 094 to − 0.20***

Results are presented as p values showing the significance of each relationship across all conditions (all data collapsed by condition and time), between COLD/TN (collapsed by condition and time) and within conditions. Time was included as a factor in all models. ES is not presented as this would involve crossover between statistical methods. Significant relationships and effects of time are highlighted in bold and italicized.

### Skin temperature

Tsk (see Fig. 2 and Table 4) increased in HOT [n = 9, + 6.5 (5.19–7.74) °C; p < 0.0001; ES: 9.3, ‘very large’] and decreased in COLD [n = 7, − 2.38 (− 3.2 to − 1.75) °C; p < 0.0001; ES: 5.2, ‘very large’] but did not change in TN [n = 9, + 0.76 (− 0.65 to 4.3); p = 0.0679].

**Table 4:** Mean skin temperature and thermal perception Δ changes across time.

	Time (min)							Main effects		
	BL	0	30	60	90	120		Condition	Time	Condition * time
**Tsk (°C)**							**Main effect**	***p < 0.0001***	***p < 0.0001***	***p < 0.0001***
Hot n = 9	30.51 (29.88–31.25)	5.38^#○^ (4.15–6.29)	6.23^#○^ (5.24–7.09)	6.31^#○^ (5.49–7.24)	6.36^#○^ (5.49–7.64)	6.5^#○^ (5.49–7.74)		HOT vs COLD ***p < 0.0001***		0–120 min ***p < 0.0001***
TN n = 9	32.46*^●^ (29.14–33.68)	0.14*^○^ (− 0.25 to 0.1)	0.5*^○^ (− 0.75 to 3.9)	0.73*^○^ (− 0.15 to 4.25)	0.7*^○^ (− 0.45 to 4.35)	0.76*^○^ (− 0.65 to 4.3)		HOT vs TN ***p < 0.001***		p ≥ 0.0679
Cold n = 7	30.57^+●^ (29.67–31.6)	− 0.08^+▲^ (− 0.45 to 0.2)	− 1.82^+○^ (− 1.25 to − 2.3)	− 2.13^+○^ (− 1.4 to − 2.25)	− 2.37^+○^ (− 1.6 to − 3.3)	− 2.38^+○^ (− 1.75 to − 3.2)		TN vs COLD ***p < 0.0001***		15–120 min ***p ≤ 0.0016***
**TS** **n = 9**							**Main effect**	***p < 0.0001***	p = 0.4612	***p = 0.0022***
Hot		5.61^#○^ (4.0–6.0)	0.44^#●^ (0.0–2.0)	0.67^#●^ (0.0–2.0)	0.61^#○^ (0.0–2.0)	0.67^#○^ (0.0–2.0)		HOT vs COLD ***p < 0.0001***		60–120 min ***p ≤ 0.0201***
TN		4.78*^▲^ (4.0–6.0)	− 0.17*^▲^ (0.0 to − 1.0)	− 0.17*^●^ (0.0 to − 1.0)	− 0.28*^●^ (0.0–to 1.0)	− 0.28*^●^ (0.0 to − 1.0)		HOT vs TN ***p < 0.0001***		p ≥ 0.2428
Cold		3.22^+●^ (3.0–4.0)	− 0.44 (0.0 to − 1.0)	− 0.61 (0.0 to − 2.0)	− 1.17^+●^ (0.0 to − 2.0)	− 1.11^+●^ (0.0 to − 2.0)		TN vs COLD ***p < 0.0001***		60–120 min ***p ≤ 0.0201***
**TC** **n = 9**							**Main effect**	***p < 0.0001***	p = 0.9404	***p = 0.0067***
Hot		4.94^#○^ (4.0–6.0)	0.06 (− 0.5 to 1.0)	0.44^#▲^ (0.0–2.0)	0.67^#●^ (− 0.5 to 2.0)	0.89^#●^ (0.0–2.0)		HOT vs COLD ***p < 0.0001***		90–120 min ***p ≤ 0.0350***
TN		4.22*^▲^ (4.0–5.0)	0.33 (0.0–1.0)	0.44 (0.0–1.0)	0.33 (0.0–1.0)	0.44 (0.0–1.0)		HOT vs TN P = 0.5174		p ≥ 0.1586
Cold		3.0^+●^ (2.0–4.0)	− 0.28^+▲^ (0.0 to − 1.5)	− 0.39^+▲^ (0.0 to − 1.5)	− 0.78^+●^ (0.0 to − 2.0)	− 1.0^○+●^ (0.0 to − 2.5)		TN vs COLD ***p < 0.0001***		90–120 min ***p ≤ 0.0141***
**SHV** **n = 9**							**Main effect**	***p < 0.0001***	***p = 0.0006***	***p < 0.0001***
Hot		1.0 (1.0–1.0)	0.0 (0.0–0.0)	0.0^#▲^ (0.0–0.0)	0.0^#●^ (0.0–0.0)	0.0^#○^ (0.0–0.0)		HOT vs COLD ***p < 0.0001***		p = 1.0000
TN		1.0 (1.0–1.0)	0.0 (0.0–0.0)	0.0 (0.0–0.0)	0.0 (0.0–0.0)	0.0 (0.0–0.0)		HOT vs TN p = 1.0000		p = 1.0000
Cold		1.0 (1.0–1.0)	0.22 (0.0–1.0)	0.44^+▲^ (0.0–1.0)	0.89^+●^ (0.0–2.0)	0.94^+○^ (0.0–2.0)		TN vs COLD ***p < 0.0001***		45–120 min ***p ≤ 0.0074***

Values expressed as mean (range). Raw BL values presented for context thermal perceptions (1–7 scale) were only recorded during the experimental period (0–120 min)]. Results are presented every 30 min for clarity. Symbols are used to denote significant between condition interactions (e.g. *Hot vs TN, ^#^Hot vs Cold, ^+^TN vs Cold) and the ES (e.g. ^■^‘trivial’, ^□^‘small’, ^▲^‘moderate’, ^●^‘large’, ^○^‘very large’). The condition*time column exhibits at what time points a significant effect occurred within condition until the significant effect was lost (e.g. Tsk in HOT was significantly different from BL from 0–120 min). Significant main effects are highlighted in bold and italicized.

### Thermal perception

Increases in TS (+ 0.67 (0.0–2.0); p = 0.0201; ES: 0.9, ‘moderate’) and TC (+ 0.89 (0.0–2.0); p = 0.0051; ES: 1.0, ‘moderate’) were observed in HOT (n = 9) and decreases in COLD (n = 9, TS: − 1.11 (− 2.0 to 0.0); p = 0.0001, ES: 1.4, ‘large’; TC: − 1.0 (− 2.5 to 0.0); p = 0.0017; ES: 1.1, ‘moderate’). There was no change in TS [− 0.28 (− 1.0 to 0.0); p = 0.3301] or TC [+ 0.44 (0.0–2.0); p = 0.1586)] observed in the TN (n = 9). SHV increased in the COLD (n = 9, + 0.94 (0.0–2.0); p < 0.0001; ES: 1.5, ‘large’). Perceptions of SHV did not change from baseline (‘not at all shivering’) in HOT or TN (n = 9, p = 1.0000). For main effects, interactions and ES see Table 4.

## Discussion

HOT [~ + 0.8 Trec (0.4–1.2) °C; ES: 2.9, ‘very large’] and COLD [~ − 0.5 Trec (− 0.8 to − 0.2) °C; ES 2.6, ‘very large’] disrupted Trec homeostasis (i.e. Trec alterations exceeded the stated meaningful change; ± 0.3°C^39^) at rest compared to baseline whilst TN did not [+ 0.1 (− 0.2 to 0.4 °C)]; as hypothesized in HOT [hypothesis (i)] and TN (iii) but not COLD (ii). COX1 was not detected at any time point within condition whilst changes in COX2 were seen in COLD [− 7.0 (− 16.52 to 3.65) ng ml^−1^; p = 0.0033; ES: 1.0, ‘moderate’]; rejecting hypothesis (iv) and (v) respectively. PGE2 did not change significantly at any time point within condition compared to baseline; rejecting hypothesis (vi). Significant relationships between COX2/PGE2 in COLD (p = 0.0012), COX2/PGE2 (p = 0.0243) and PGE2/Tc (p = 0.0079) in COLD/TN [(collapsed by condition and time) NB: results from this analysis should be tempered given the difference in environmental temperature between conditions] were seen, with higher PGE2 concentrations associated with higher Trec compared to baseline, supporting the premise of a COX/PGE2 thermogenic pathway as hypothesized here and elsewhere^19,35^. The nuances of these biochemical responses and changes in Trec regulation will subsequently be discussed.

Given the robust evidence from rodent models [simultaneous reductions in Tc (up to 3.9 °C) and PGE2 (96%)^20,21,36^] Trec changes were hypothesized to be underpinned by the COX1/PGE2 biochemical pathway. Theoretically, COX1 is expressed constitutively (i.e. in an afebrile state) and performs ‘housekeeping’ functions to maintain homeostasis^51,52^ thus, would be the catalyst for increases in PGE2 and thus rises in Tc. However, COX1 was not detected [despite the high sensitivity (0.225 ng ml^−1^) of the ELISA kit employed] and within the present design it appears not to behave in the manner inferred in humans^19,35^ regarding homeostatic Tc control. COX2 is widely accepted as an inducible form of COX and is upregulated in pathological states (i.e. a febrile state^33,34^). However, local (forearm) COX2 inhibition (celecoxib) has attenuated sweat response during exercise under heat stress in healthy (free from pathology/febrile state) humans^53^. Further, COX2 mRNA has been present in many tissues free from pathology (e.g. lung, liver, kidney, stomach^54–57^) but this is not conclusive evidence of constitutive COX2 expression; mRNA expression of COX2 does not always guarantee the presence of a functional COX2 enzyme^57,58^. It is generally accepted that in a febrile state it is COX2 that is upregulated (i.e. responds to the febrile stimulus) inducing an increase in the pyrogenic mediator PGE2 and subsequently a rise in Tc (i.e. thermogenesis^24,25^). The current study aimed to identify the central mechanism of Tc regulation by COX/PGE2. Interestingly, a series of studies has shown that COX/PGE2 may be implicated in peripheral mechanisms that may modulate Tc^53,59–61^. Indeed, during exercise heat stress (35 °C 20% RH) inducing moderate (400 W) metabolic heat production local (forearm) COX inhibition reduced sweating but not cutaneous vasodilation; this attenuation of sweating was however lost during a bout of high (700 W) metabolic heat production^53,59^. Somewhat paradoxically local administration of COX products PGE1, PGE2 and prostacyclin (or PGI2) do not appear to influence sweating but do induce cutaneous vasodilation^60,61^; although the thermoneutral conditions in these studies^60,61^ may account for this superficial paradoxicality relative to other data^53,59^. Ultimately, the COX/PGE2 pathway is highly complex, likely integrating central and peripheral mechanistic effects to govern dynamic whole body thermoregulation. In HOT, COX2/PGE2 concentrations did not change, indicating the rise in Trec (all participants in HOT ≥  + 0.3 °C Trec change; indicative of an uncompensable hot environment) was not a product of the COX/PGE2 pathway; whether a larger increase in Trec would have seen such a response requires further investigation.

COX1 was not detected in this study. However, COX2 significantly decreased in COLD and TN indicating catalysis of readily available (i.e. constitutively expressed) COX2 and a thermogenic response (i.e. utilization of COX2 inducing upregulation of PGE2 thus heat production and defense of Trec). However, caution must be exercised when interpreting any in vivo molecule/marker in isolation within a host as complex as humans. Indeed, solely attributing homeostatic ‘housekeeping’ functions to COX1 and pathophysiological functions to COX2 oversimplifies the role of these highly complex isoforms in humans and in some instances is wholly inaccurate^33^. Indeed, it is estimated ~ 50% of human genes generate multiple mRNA products, often unproductive targets for degradation^32^. COX1 and COX2 oxidize arachidonic acid at different concentrations, > 10 µM and ≤ 2.5 µM respectively^62,63^. It is likely therefore, that COX1 oxidizes arachidonic acid in the immediate response to pathological stimuli (i.e. the febrile response) where intense activation of phospholipases release a burst of arachidonic acid, COX2 only becomes functional as arachidonic acid concentration falls below the threshold of COX1 oxidation^26^. COX2 is inducible across all mammalian cells^64^ and when both isoforms are available in the same cell, use of exogenous arachidonic acid is via COX2^62^. At arachidonic acid concentrations between 50 nM and 1 µM, COX1 produces less than 25% of the ‘product’ of COX2^65^. Importantly this concentration range is likely what is available in vivo and allows COX2 to act independently of COX1^64^. Furthermore, COX isoforms are the rate limiting enzyme for a plethora of prostaglandins [(PGE2, PGD2, PGF2α and PGI2 and thromboxane’s; TXA2 and TXB2^52^]. COX oxidization of arachidonic acid produces the unstable prostaglandin intermediary PGG2 that is peroxidized to PGH2; metabolism of this compound via specific enzymatic activities produces the required PG^52^. The abundance of prostaglandin’s that can be produced from COX oxidation of arachidonic acid dictate that the production of PGE2 is not directly related to the concentrations of COX utilized (i.e. one ‘unit’ of COX does not produce one ‘unit’ of PGE2). Together, such data may in part, underpin the lack of detectable COX1 in the current study.

Nevertheless, the data from COLD and the individual variation in Trec in TN [+ 0.1 (− 0.2 to  + 0.4) °C; ~ 33% participants having a change in Trec that exceeded the ≥ 0.3 °C meaningful change in Trec^39^] questions whether the TN condition was truly thermoneutral for all participants [i.e. changes in metabolic heat production/evaporative heat loss and Trec should not have been observed using the TN environmental conditions (TN: 30 °C 40% RH) recommended widely; 28–32°C^66–68^]. It is plausible given the demographics of the participant’s [residing in a hot desert climate (yearly: mean: 28 °C; mean high: 42 °C; mean low: 14 °C) and their regular exercise heat stress exposure; such implications are discussed in detail within the next paragraph] generic models of the TNZ may not in retrospect be applicable to this cohort. Recent conjecture suggests much individual variation (23–26°C^40^; ~ 26–33°C^69^) in TNZ boundaries across populations; indeed, one study was not able to distinguish an upper critical temperature limit for the TNZ using a dynamic approach (lower limit: ~ 23°C^70^). However, without measurement of skin blood flow (SkBF) to determine changes in vasoconstriction/dilation or the assessment of metabolic rate and sweat rate within the present design, absolute certainty regarding these postulations is not possible. Therefore, the TN condition may have induced mild cold stress in some of the current cohort although, a significant upregulation of PGE2 and increase in Trec (as hypothesized elsewhere^19^) did not occur in either TN or COLD. However, there was a significant relationship between COX2 and PGE2 in COLD and COLD/TN (collapsed by condition and time) where higher concentrations of PGE2 were associated with higher Trec (see Fig. 4 panel E and H). This data suggests a thermogenic contribution from the COX/PGE2 pathway which may however be compromised within the current cohort (discussed in detail in the paragraph below) relative to its ability to evoke a thermogenic increase in Trec [seen elsewhere^22,23^ in identical conditions (20 °C 40% RH)].

The participant demographic in this study may have significant implications for the interpretation of the results. In COLD it was hypothesized that Trec would be defended (i.e. no significant change in Tc), as seen elsewhere when participants were exposed to the same (20° C 40% RH) or colder (10 °C) conditions and thermogenic mechanisms were not inhibited (e.g. via pharmaceutical intervention^22,23^). The participants in the current study resided in a hot climate (Doha, Qatar) and regularly exercised outside. This study took place in the summer months (May–July; average temperature 33 °C with peaks of ~ 42 °C) likely resulting in some heat acclimatization adaptations, although this study did not quantify this. It is well noted that cold/heat acclimation/acclimatization elicits a variety of thermoregulatory adaptations in humans^2,14^, other mammals^71^ and rodents^72^. Indeed, in response to a 5-h cold exposure (− 5 °C) cold acclimated rat Tc increased (+ 1 °C) yet decreased (− 11 °C) when heat acclimated^73^. A previous human study utilizing non-heat acclimatized individuals saw Tc reduced (up to 0.39 °C) in the same cold conditions as the current study [20 °C 40% RH (by 0.57 °C in 10 °C 40% RH)] only when ACT was administered (a potent COX inhibitor and hypothermic agent) but remained stable in the placebo condition [compared to a maximum − 0.8 °C reduction in Trec in the current study (COLD: 20 °C 40% RH) without ACT administration]^22,23^. In the only human study to date assessing COX2 and PGE2 changes following a heat acclimation protocol, COX2 (17.3%) and PGE2 (18.5%) concentrations decreased from pre to post acclimation^74^. Plasma volumes (that are significantly altered in response to heat acclimation^2^) were not adjusted relative to the ELISA analysis performed within that study^74^ and the acclimation protocol only involved 10 half-body water immersions (42 °C) of 30 min across 3 weeks; so the data from that study^74^ should be interpreted with some caution. To further illustrate the effect of heat acclimation/acclimatization status on the study specific cold stress response experienced (e.g. the present data compared to^22^), identical conditions (20 °C 40% RH) were perceived as colder (mean TS score 2.1 = ‘cool’) in the present study compared to previous data (mean TS score 3.4 = ‘comfortable’^22^). Together, these results present a plausible explanation for the heat acclimated rodent response to a cold exposure^73^ and the inconsistencies presented between our and previous human data^22,23^. Finally, air flow is a central component of the conceptual heat balance equation and in the present study air flow was minimal (although not recorded) whilst being unreported elsewhere^22,23^; future work should control and report this variable. The data in the current study [increase in PGE2 = higher Trec in COLD and COLD/TN (collapsed by condition and time)] and discussion presented here posit that the COX/PGE2 pathway may be compromised by heat acclimation/acclimatization, rendering rodents^73^ and humans^74^ less able to defend their Tc in response to cold stress. The lack of biochemical analysis in the previous human data^22,23^ and quantifiable acclimation status in the current study however do not allow definitive conclusions regarding these postulations. Therefore, further deductive human research designs comparing heat acclimatized and non-acclimatized participants are required to explore the above discussions.

The disparity between the human data presented in the current study and rodent models may be due to the differences between species (e.g. coverage of hair and body size) which significantly affects how each thermoregulate^75,76^. For example, humans heavily rely on evaporation for heat loss, particularly during exercise^77^, whereas rodents easily dissipate heat via passive mechanisms because they have a large surface area to volume ratio^76^. As discussed previously, acclimation/acclimatization affects thermoregulatory mechanisms^2,14,72^; rodents used for research are generally housed in environments that are below their TNZ^72,78^. Constant exposure to this thermal cold stress (i.e. a cold environment below their TNZ) induces compensatory mechanisms/cold acclimation (i.e. increases in non-shivering thermogenesis and metabolic activity^72^); as seen in the rodent literature discussed above^20,21,36^. Whether the COX/PGE2 thermogenic pathway exhibited in rodents is therefore a product of chronic cold stress requires further deductive investigation. Importantly, the rodent evidence for the COX/PGE2 pathways influence on thermoregulation is strong^20,36^ but not unequivocal. Indeed, recent research has cited that inhibition of lipolysis and mitochondrial function could be the cause of the hypothermic actions of ACT in afebrile rodents and not the inhibition of COX and PGE2^79^. The data discussed here highlight it is paramount that results from rodent models are not generalized to humans without investigation^31^ and further in vivo research is required to elucidate the thermoregulatory influence of the COX/PGE2 pathway in humans.

Future work would benefit from addressing the limitations within the present experimental design. Normative values for COX/PGE2 in any human bodily fluid (e.g. blood, cerebrospinal fluid) would aid in the interpretation of these and future results and determine if blood concentrations reflect those in the brain and can be used as a reliable measure of central Tc regulation. Further, there is a need to understand central and peripheral COX and PGE2 thermoregulatory mechanisms and identify if sex differences in the response of COX/PGE2 to environmental temperature exist. Quantifiable measures of shivering, sweating, heart rate and SkBF would have been advantageous to understand the thermoregulatory responses and the level of thermal stress elicited by the environmental temperatures relative to the TNZ. Additionally, given its major role in thermoregulatory responses to extremes of environments either side of the TNZ, acclimation status (both hot and cold) should be well-controlled and/or experimentally manipulated within future research designs.

## Conclusion

To conclude, there was a significant relationship between COX2 and PGE2 in COLD and COLD/TN (collapsed by condition and time) and higher Trec was associated with higher PGE2 concentrations. Further deductive research designs are required to elucidate the thermogenic influence of this pathway in humans alongside the precise implication of acclimation (hot and cold) on COX/PGE2 concentrations and Tc within this paradigm.
